# Future intensification of extreme Aleutian low events and their climate impacts

**DOI:** 10.1038/s41598-021-97615-7

**Published:** 2021-09-15

**Authors:** K. Giamalaki, C. Beaulieu, S. A. Henson, A. P. Martin, H. Kassem, D. Faranda

**Affiliations:** 1 Ocean Sciences Department, University of California, Santa Cruz, CA USA; 2 National Oceanography Centre, European Way, Southampton, UK; 3 Ocean and Earth Science, University of Southampton, European Way, Southampton, UK; 4 Laboratoire des Sciences du Climat et de l’Environnement, LSCE/IPSL, CEA- CNRS-UVSQ, Université Paris-Saclay, Gif-sur-Yvette, France; 5 London Mathematical Laboratory, 8 Margravine Gardens, London, W6 8RH UK; 6 LMD/IPSL, Ecole Normale Superieure, PSL Research University, Paris, France

**Keywords:** Climate sciences, Ocean sciences

## Abstract

Extreme Aleutian Low (AL) events have been associated with major ecosystem reorganisations and unusual weather patterns in the Pacific region, with serious socio-economic consequences. Yet, their future evolution and impacts on atmosphere–ocean interactions remain uncertain. Here, a large ensemble of historical and future runs from the Community Earth System Model is used to investigate the evolution of AL extremes. The frequency and persistence of AL extremes are quantified and their connection with climatic variables is examined. AL extremes become more frequent and persistent under the RCP8.5 scenario, associated with changes in precipitation and air temperature patterns over North America. Future changes in AL extremes also increase the variability of the sea surface temperature and net heat fluxes in the Kuroshio Extension, the most significant heat and energy flux region of the basin. The increased frequency and persistence of future AL extremes may potentially cause substantial changes in fisheries and ecosystems of the entire Pacific region as a knock-on effect.

## Introduction

The Aleutian Low (AL) pressure system is a major climatic feature in the North Pacific, formed over the Aleutian Islands during boreal winter. The AL affects the weather and climate of North America and Eurasia, significantly impacting temperature and wind patterns^1,2^. Changes in the AL frequency and intensity may also result in anomalous precipitation events over Pacific Asia and the west coast of the United States^1,3^. The intensified AL leads to a strong high-pressure ridge over the west coast, associated with very low precipitation years over the area^4,5^. AL extreme variability has been associated with fluctuations in fisheries in the eastern North Pacific (e.g.^6–8^) and with extensive marine ecosystem reorganizations, such as the regime shift in the late 1970’s^7,9,10^.

The magnitude of the AL pressure anomalies and the duration of AL events affect the North Pacific ocean conditions by altering the wind stress curl and wind speed (e.g.^11–13^), with knock-on effects on sea surface temperature (SST), sea surface height and net heat flux. Anomalous SST and sea surface height in the north-eastern Pacific caused by extreme AL events propagate towards the western basin through Rossby waves, with the signature becoming evident in the Kuroshio region with a lag of 3–4 years^10,14,15^. The Kuroshio Extension region is the area of maximum interactions in the North Pacific in terms of heat and momentum feedback to and from the atmosphere^16–18^. The Kuroshio Extension SST and net heat flux variability both drive, and are also significantly driven by, the North Pacific atmospheric circulation^19,20^.

Fluctuations of the AL are recognized as one of the main sources of variability in the North Pacific climate system^21^. The AL has been identified as the main driver of the Pacific Decadal Oscillation (PDO;^22–24^) and is teleconnected with the tropical El Niño–Southern Oscillation (ENSO;^22,25^). In fact, the AL intensifies in response to strong ENSO events resulting in a positive PDO pattern with warmer than usual north-eastern Pacific SST^25,26^. ENSO and its associated SST variability in the North Pacific have been also considered a precursor to changes in precipitation^27,28^ and surface air temperature (SAT) patterns^29^ over the west coast of the United States. Multiple studies of future climate projections suggest that El Niño events will become more frequent in a warming climate^30–33^, with a potential intensification of the AL^34^. Future changes to AL extremes are reflecting climate change and are expected to be important because of its significant role in shaping the hydroclimate in North America and affecting the North Pacific physical and ecological dynamics.

As the regulating mechanisms of the AL (i.e. ENSO teleconnections) intensify in the ‘business-as-usual’ future RCP8.5 scenario, the AL and its subsequent effects are likely to increase. Previous studies have examined the consequences of a warming scenario on the North Pacific mean state, the AL variability, and the El Niño teleconnections^26,34,35^. However, the future frequency of extreme AL events and their oceanic and atmospheric response still remain unclear. Here, we assess changes in AL extreme events by comparing the intensity and frequency of North Pacific extreme SLP patterns in past and future simulations of the Large Ensemble of the Community Earth System Model version 1 (CESM1-LENS^36^). We show that extreme AL events become stronger and more frequent under the RCP8.5 scenario. To consider wider impacts due to future changes of the AL, its relationship with precipitation and SAT over North America, and the SST and net heat flux over the North Pacific is examined. We quantify the oceanic and atmospheric response that follows the atmospheric extremes by evaluating the change in the dominant period of common variability of the AL SLP and each one of these climate parameters.

## Results

### Increased persistence and frequency of future Aleutian Low extreme events

**Figure 1:**
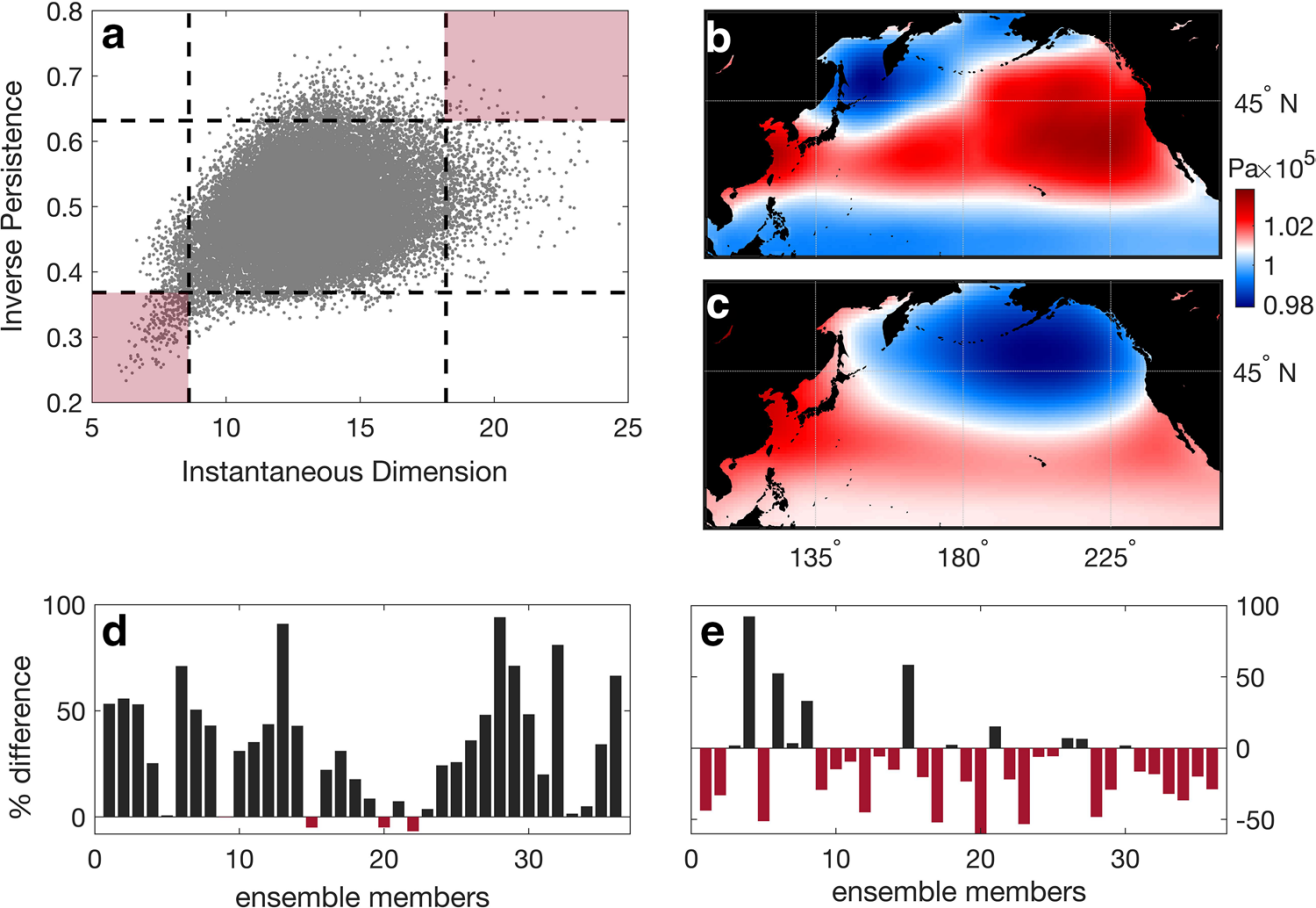
The dynamical indicators for both the historical and RCP8.5 ensembles of the CESM1-LENS calculated to detect the extreme SLP events. (**a**) Example of daily dynamical properties for one ensemble member (member 16). Black dashed lines delimit the lower 2% and upper 98% percentiles of the dynamical properties. The lower percentile of the properties represents increased persistence and low frequency, whereas the higher percentile signifies decreased persistence and high frequency of the daily North Pacific SLP patterns. The red shaded quantiles represent the most extreme cases for both properties. (**b**) Average conditions of daily North Pacific SLP points in the extreme high quantile for the example ensemble member. (**c**) Same as (**b**) for the extreme low quantile. (**d**) Percentage difference between the numbers of points (daily North Pacific SLP) falling within the extreme low quantile in the historical runs and the future RCP8.5 scenario simulations for each ensemble member. Red bars represent a decrease in the number of the daily North Pacific SLP points occurring in the extreme low quantile for both inverse persistence and instantaneous dimension, which describes more stable North Pacific atmospheric patterns. Black bars represent an increase in the number of points occurring in the extreme low quantile for both properties. (**e**) Same as (**d**) but bars represent the points of the extreme high quantile for both properties, which represent the most transient phases of the North Pacific SLP. Map figures were created using Matlab^38^.

To quantify the persistence and frequency of extreme AL events, we use dynamical indicators to describe the dynamical state of the system (see “Methods”). Specifically, the inverse persistence indicates how a daily SLP pattern persists through time, whereas the instantaneous dimension represents the predictability and repeatability of that pattern throughout the time-series. The two dynamical indicators calculated for the future simulation of one example ensemble member (CESM1-LENS member 16) are used here for illustration purposes and are displayed in Fig. 1a. The two most extreme areas of the scatterplot (two red shaded upper (0.98) and lower (0.02) quantiles in Fig. 1a) represent the North Pacific daily SLP configurations that present extremely high and extremely low estimations for both dynamical indicators. The average of the points within the quantile of the extreme high dynamical properties, which represents conditions of low stability and predictability, displays a transitional North Pacific blocking pattern (Fig. 1b for the example member). This is consistent with the results found by Faranda et al.^37^ for the North Atlantic, where blocking patterns were also associated with low persistence and high predictability. On the other hand, the extreme low quantile represents a deepened AL pattern (Fig. 1c for the example member) and signifies increasing frequency and persistence of the pattern in the region. The deepening of the AL, in terms of magnitude, is also apparent when comparing the frequency distributions as well as the lowest 2% percentiles of the spatially averaged monthly AL SLP time-series of the historical simulations and the future RCP8.5 runs (Supplementary Information, Fig. S1). Furthermore, an increase of points with extremely low dynamical properties in the future runs compared to the past is shown in 92% (33 out of 36) of the total of the ensemble simulations (Fig. 1d). The number of points (*i.e.* days of North Pacific SLP) falling within this extreme high quantile decreases in the future (Fig. 1e) in approximately 70% (25 out of 36) of the ensemble members.

**Figure 2:**
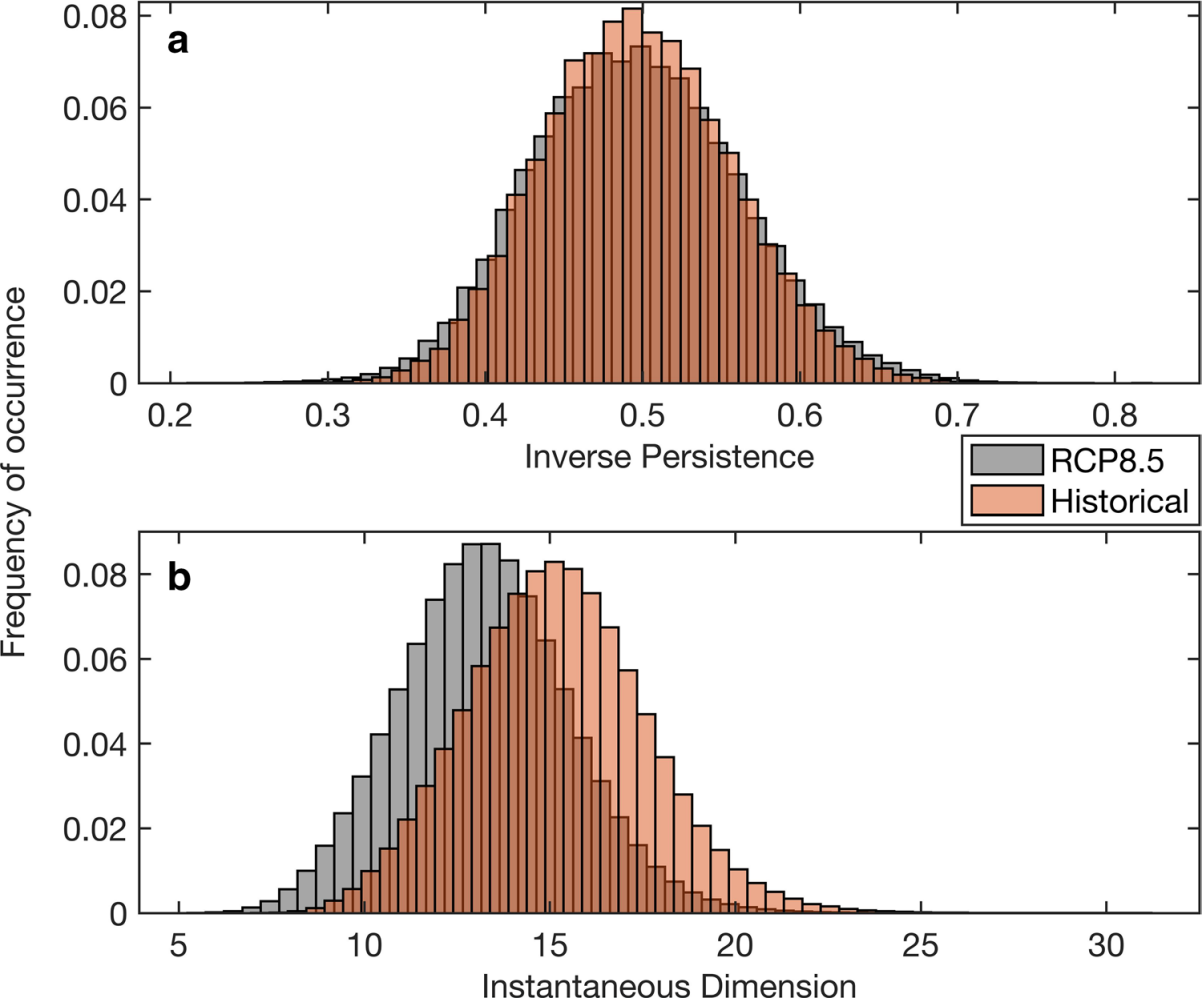
The dynamical indicators for the historical and RCP8.5 ensemble runs of the CESM1-LENS (whole distribution). (**a**) The inverse persistence and (**b**) the instantaneous dimension of all the ensemble members in the historical (orange) and in the future RCP8.5 simulations (grey).

The dynamical indicators for the historical and RCP8.5 ensemble runs of the CESM1-LENS are presented in Fig. 2 (whole distribution, rather than just 2nd and 98th percentiles). The extension of both the upper and lower tails of the distribution of the inverse persistence indicates that future extreme North Pacific SLP patterns have an increased variability of their residence time in the area (Fig. 2a). A lower inverse persistence means that the dynamical system trajectory is slow leaving the neighborhood confined by one point^39^, and describe more stable dynamic fields that tend to have slower variations^40^. This means that when an SLP pattern emerges, it is more likely to persist in the region for a longer period in the future projections compared to the past simulations. The slight extension of the lower tail of the histogram in Fig. 2a suggests that the persistence of the stable SLP configurations governing the North Pacific (i.e. Aleutian Low and North Pacific High) increase under the RCP8.5 scenario. Similarly, extremely unstable SLP patterns, that are represented by trajectories rapidly leaving the neighborhood around one point, are equivalently increasing in the RCP8.5 scenario. The elongated upper tail of the inverse persistence histogram (Fig. 2a) indicates that these unstable SLP patterns in the area (*i.e.* spring transition pattern and North Pacific blocking pattern) will become more transient and more likely under global warming. On the other hand, a substantial shift occurs in the instantaneous dimension of the whole North Pacific SLP system (Fig. 2b). A low instantaneous dimension of a given atmospheric pattern suggests a higher likelihood for the pattern to emerge again in the system^37^, which describes the rarity of the daily SLP configuration examined in each time-step^40^. In other words, a lowering of the dimension indicates that the most unstable patterns will not be favored in the future climate and that the atmospheric circulation in this area will be more predictable. The lowering in dimension found here suggests that the dominant SLP patterns (*i.e.* a wintertime Aleutian Low and a summertime North Pacific High) occurring in the North Pacific will be more frequent in the future compared to unstable transitional patterns (*i.e.* the North Pacific blocking and the spring transition patterns). These results are pointing towards an increasing stability of atmospheric motions that are coherent with those found for the Atlantic basin^41^. The distribution differences in both indicators were tested with the two-sided Kolmogorov-Smirnoff test, suggesting significant distribution differences in each case (significance level of 99%).

### Relationship of the intensified Aleutian Low with weather patterns over North America

**Figure 3:**
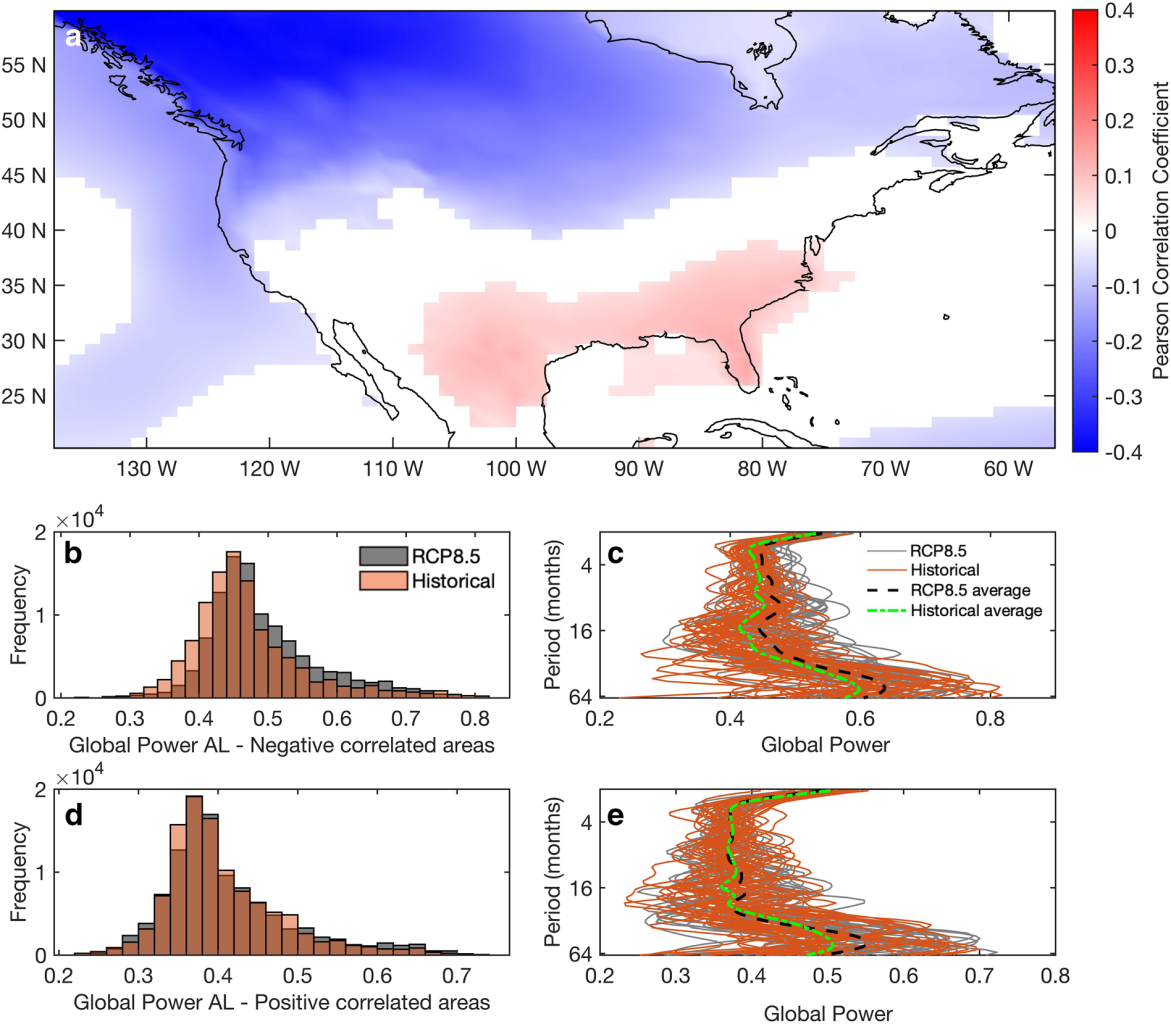
(**a**) Point-wise Spearman correlation coefficient between the spatially averaged historical and RCP8.5 AL SLP and SAT in north America (**b**) The frequency distribution of the global power of wavelet coherence between the past and future simulations of the negatively correlated areas (shown in (a)) between the spatial average of the AL SLP over the area 45°–65° N, 160° E–140  W and surface air temperature over North America. (**c**) The global power of the 36 past (orange lines) and the future (grey lines) ensemble members. Black and green dashed lines represent the average global power of future and past multi-ensemble members respectively. (**d**) Same as (**b**) but for positively correlated areas. (**e**) Same as (**c**) but for positive correlations between AL SLP and SAT. Map figure was created using Matlab ^38^.

Due to its controls on atmospheric circulation, changes in the AL have the potential to impact weather patterns over North America. Here we examine the possible effects on surface air temperature (SAT) and precipitation. Regions where changes in the AL SLP significantly affect SAT over North America are presented in Fig. 3a. Atmospheric circulation patterns (e.g. anomalous winds) related to a deepened AL have been linked to warming trends over Canada and Alaska^42,43^. A negative correlation between the AL SLP and the SAT is predominant in most of the northern part of North America, which is stronger in the northwest. Contrastingly, southeastern North America is shown to be slightly positively correlated to the AL SLP.

The coherence (see “Methods”) between past and future simulations of the AL SLP and SAT is estimated in order to assess the change in the relationship between the fields at different periods. The frequency distribution of the global power of the wavelet coherence (i.e. distribution over frequencies averaged in time) between negatively correlated areas of AL SLP and SAT presents a shift towards higher power in the future members over all the periods considered (Fig. 3b) (Kolmogorov-Smirnoff tests, 99% significance level; Supplementary Information, Figure S2). The intensified and more frequent extreme AL strengthens basin-scale winds resulting in warmer SAT over the west coast of North America^44,45^. Our results show an intensification of this relationship under future scenarios. The global power of individual past and future ensemble members follow different patterns throughout the multiple periods (Fig. 3c), emphasizing the influence of internal variability of the system, since the simulations are constrained by the same historical and RCP8.5 radiative forcing^44^. Still, the average global power of the wavelet coherence between AL SLP and SAT over North America is higher in the future compared to the past, during the periods between 4 and 40 months (Fig. 3c). This shift towards higher average global power suggests the enhanced influence of the future AL on the North American SAT (negatively correlated areas in Fig. 3a).

The differences between the frequency distributions of the global power of the wavelet coherence in the past and future simulations, as well as their average global power, were both significant (Kolmogorov-Smirnoff test, 99% significance level; Supplementary Information, Figure S2), however only minor discrepancies are noticeable between them (Fig. 3d,e). Although possible teleconnections with the AL may play some role, other mechanisms (e.g. the influence of ENSO and North Atlantic Oscillation) may be more important in driving the SAT variability over southeast North America^46^.

**Figure 4:**
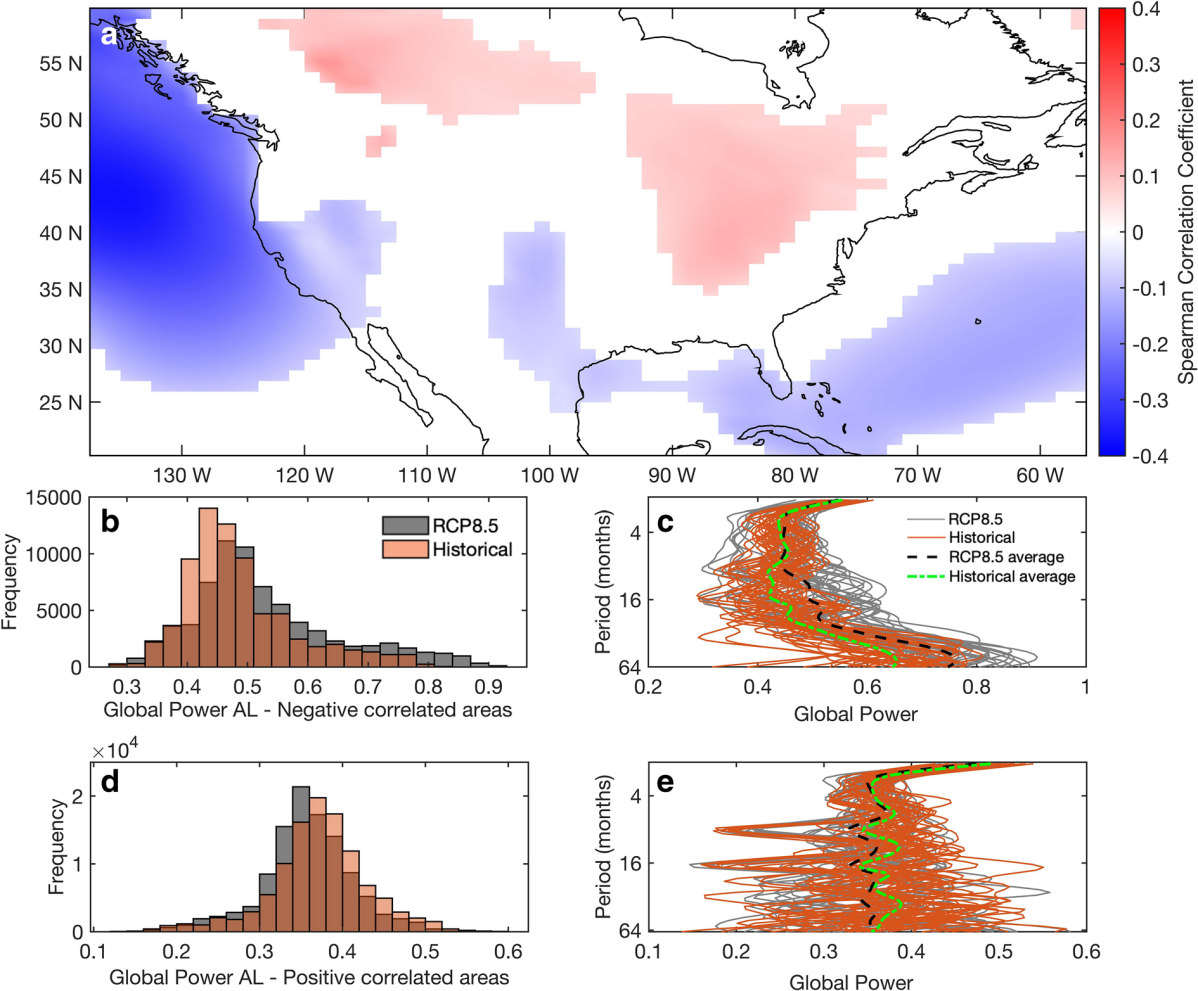
(**a**) Point-wise correlation coefficient between the spatially averaged AL SLP and precipitation in North America. (**b**) The frequency distribution of the global power of wavelet coherence between past (orange bars) and future (grey bars) simulations of negatively correlated areas (shown in 4a) between the spatial average of the AL SLP over the area 45°–65° N, 160° E–140 W and precipitation over North America. (**c**) Global power of the 36 past (orange lines) and the future (grey lines) ensemble members. Black and green dashed lines represent the average global power of future and past multi-ensemble members respectively. (**d**) Same as (**b**) but for positively correlated areas. (**e**) Same as (**c**) but for positive correlations between AL SLP and precipitation. Periods over 64 months have been removed due to ‘cone of influence’ effects. Map figure was created using Matlab^38^.

A deepened AL has been related to precipitation over North Pacific^3^ and to the precipitation dipole over the U.S. west coast^5^. Specifically in California, an intensified AL increases the precipitation extremes through strengthened atmospheric vapor and enhanced atmospheric rivers^47^. The correlation between the AL SLP and precipitation over North America is presented in Fig. 4a, where precipitation in the northwest and southeast have a negative relationship with the AL SLP and the opposite stands for areas in continental US and Canada. The frequency distribution of the global power of the wavelet coherence between negatively correlated areas of AL SLP and precipitation presents a shift towards higher power in the future members over all the periods considered (Fig. 4b). The global power of individual past and future ensemble members follows similar patterns and present increasing global power in lower frequencies indicating higher common variability of the two fields on interannual temporal scales (Fig. 4c). Furthermore, the multi-ensemble average global power for the past and future members between AL SLP and precipitation over North America highlights an intensified relationship for periods greater than 10 months (Fig. 4c). Contrary to the results for the negatively correlated areas, the frequency distribution of the global power between the positively correlated areas of AL SLP and precipitation presents a shift towards lower power in the future simulations (Fig. 4d,e). Two-sided Kolmogorov-Smirnoff tests presented consistent distribution differences in each (positive and negative correlation) case (significance level of 99%; Supplementary Information, Figure S3). The evolution of the AL and precipitation and SAT over North America over different time scales in the future has also been examined through the calculation of their common variability during a near-future (2005–2050) and a far-future (2051–2100) period under the RCP8.5 scenario (Supplementary Information, Figure S5 and Figure S6). Breaking down the global power into separate future periods highlights that the future intensification of the relationship between the AL SLP and North American precipitation and SAT occurs throughout the whole time period (as opposed to only towards the end of the century).

### Increased sea surface temperature and net heat flux in the Kuroshio Extension due to an intensified AL

**Figure 5:**
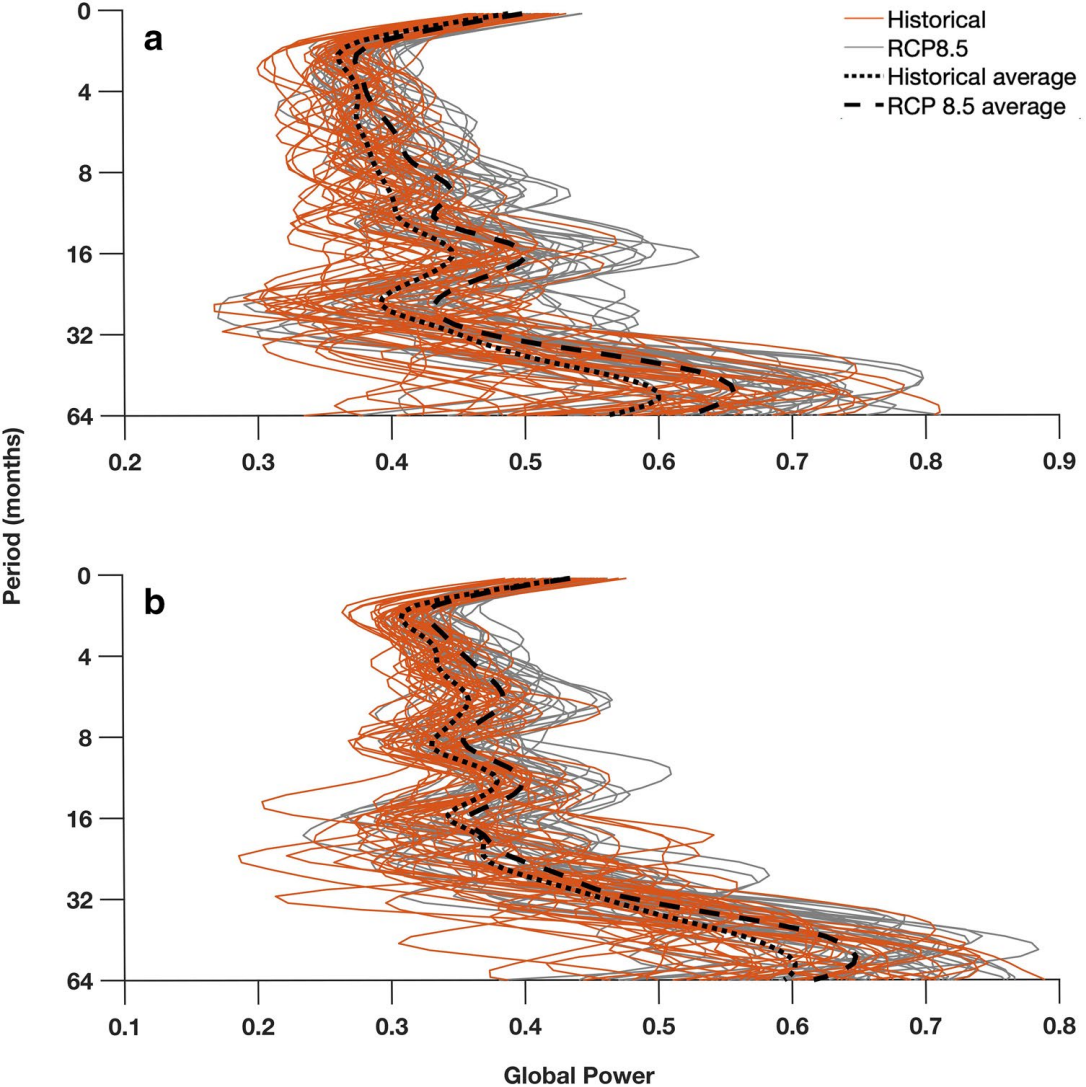
Global wavelet power (horizontal averaging in time) of the wavelet coherence of the Aleutian Low SLP in the Kuroshio Extension (**a**) net heat flux and (**b**) SST for all 36 ensemble members of CESM1-LENS in the past (orange) and in the future (grey). Black dotted and dashed lines represent the ensemble averages of the historical and RCP8.5 future runs respectively. The periods over 64 months were eliminated due to the influence of the edge effects of the wavelet transform which may produce spurious results.

The common variability of past and future simulations between the AL SLP and both net heat fluxes and SST in the Kuroshio Extension is analyzed in order to examine potential changes in their relationship at multiple frequencies. The global power of wavelet coherence of the AL SLP and the examined parameters is higher in the future members in all frequency bands (Fig. 5). It has been previously shown that the AL SLP affects the Kuroshio Extension jet and SST, as well as the variability of heat fluxes in the area^15,19,48^. Our results present the evolution of this relationship in time and highlight its intensification under the RCP8.5 future scenario. The intensified and more frequent extreme AL induce increased forcing to the Kuroshio Extension net heat flux and SST.

Although the global wavelet power patterns of the multiple ensemble members differ substantially from each other under the same scenarios (Fig. 5), on average they peak at the same frequencies (black dotted and dashed lines in Fig. 5a,b). The similar patterns on longer than annual scales indicate that the consistent mechanistic linkage between the AL SLP and the Kuroshio Extension net heat flux and SST is not altered by the internal variability of the system or the extreme variations of the individual fields (e.g. AL SLP extreme events). However, in periods shorter than the annual time scales (< 10 months) the individual ensemble members present a highly variable global power, showing that the internal variability of the system plays an important role in shaping the intra-annual relationships between the AL and SST and net hear flux. Periods larger than 64 months are not considered, due to the influence of the edge effects of the wavelet transform that become apparent in the low frequency bands. The evolution of the common variability between the AL and SST and net heat flux in the Kuroshio Extension at different time scales in the future under the RCP8.5 scenario has also been examined (Supplementary Information, Figure S7). The comparison between historical simulations, the RCP8.5 runs during 2005–2050 and the RCP8.5 for the period 2051–2100 shows that the AL and SST increase in common variability happens mostly in the far-future (2051–2100). On the other hand, the near and far future periods are equally important for the increase in common variability between the AL SLP and the Kuroshio Extension heat flux.

## Discussion and conclusions

A coupled climate model that includes physical, biogeochemical and ecosystem components was used to explore the intensification of North Pacific atmospheric extremes and their impact on atmospheric and oceanic parameters under climate change. We compared the historical runs and the ‘business-as-usual’ RCP8.5 scenario simulations of the Community Earth System Model—Large Ensemble. This analysis reveals an intensification of the North Pacific SLP under increased anthropogenic forcing, expressed as a future increase in frequency and persistence of AL extreme events.

The overall deepening of the North Pacific SLP in the future CESM1-LENS simulations and the increase of low SLP extreme events indicate that the AL pattern is strengthened under anthropogenic warming. AL variability is primarily linked to the North Pacific decadal climate variability^23,24^, which is a major source of uncertainty in the near-future model SLP projections^49^. Observational and modelling studies have demonstrated that internal variability alone can generate ENSO-like responses in the North Pacific atmospheric system^50^. Furthermore, remote connections with ENSO indirectly contribute to the AL variability via the atmospheric bridge^25^. ENSO events drive the AL SLP through nonlinear extra-tropical teleconnections triggered by the increased tropical SST anomalies^25,26^. Anomalously warm SSTs in the eastern tropical Pacific induce increased rainfall and heat from enhanced atmospheric convection. This results in upper-tropospheric divergence and vorticity which excite stationary Rossby wave trains moving poleward across the North Pacific resulting in a deepened AL^51^. Specifically, changes in the location and magnitude of ENSO SST anomalies in the equatorial Pacific alter the strength, position and persistence of the AL^34,52^. Gan et al.^34^ also suggested a deepening of the AL in the twenty-first century, stressing the importance of the effects of tropical SST anomalies. As the AL drivers (*i.e.* North Pacific internal dynamics and teleconnections to ENSO) are shown to be highly linked to anthropogenic greenhouse gas forcing^52,53^, any associated change can potentially lead to enhanced AL extreme events. The deepening of the AL SLP projected by the future simulations shown here is consistent with the intensified and more frequent ENSO events predicted under increased anthropogenic forcing^30–32,35^. It is interesting to note that, unlike the CMIP5 inter-model comparison conducted by Gan et al.^34^ associating climate model differences with natural variability, here we show that the internal variability combined with the signal of the radiative forcing alone can generate extreme changes in North Pacific SLP patterns under the RCP8.5 future emission scenario.

The changes detected in the dynamical indicators of the North Pacific SLP suggest that the persistence and frequency of SLP patterns will vary in the future. Specifically, the semi-permanent SLP patterns (i.e. Aleutian Low and North Pacific High) will become more persistent and more frequent, whereas the transitional patterns (*i.e.* North Pacific blocking and spring transition patterns) will become less stable and less frequent. These changes are likely to impact the dynamics between large-scale atmospheric fluctuations and local weather extremes^39^. Deepened AL have been related to changing weather patterns, temperature and wind field fluctuations over North America and Asia^1,2^. In response to an intensified AL and its associated basin-scale cyclonic flows, cold air temperatures, strong northerly winds and stormy conditions dominate East Asia; whereas warmer conditions caused by strong southerly winds are favored on the west coast of North America^1,3,54^. The wintertime North Pacific pressure variability also affects large-scale precipitation changes on the west coast of North America^55^. Here, we present the historical common variability between the intensified AL, SAT and precipitation both spatially and temporally and we further identify the future development of these connections. Air temperature and precipitation conditions in northwest North America are closely related to the AL variability, making them highly susceptible to changes due to an intensified and more frequent extreme AL formation in the future.

As strong and stable atmospheric patterns more commonly emerge in the North Pacific, an intensification of the oceanic response is likely to occur. The AL SLP variability and its extreme deepening have been linked to the Kuroshio Extension net heat flux and SST fluctuations^10,15,19^. The AL controls the variability of the physical parameters in the Kuroshio Extension through basin-scale changes in wind and sea surface height^19,56^. An extreme deepening of the AL increases the westerlies and causes anomalous positive wind stress curl in the central North Pacific^15^. This enhances the southward Ekman drift^11^ and produces negative sea surface height anomalies in the central North Pacific. The westward propagation of these anomalies through baroclinic Rossby waves cause lagged responses of the SST of approximately 3–4 years and further destabilize the dynamical state of the Kuroshio Extension system^14,15^. The SST fluctuations in the region generate anomalous heat fluxes^19^, resulting in an area of maximum ocean–atmosphere heat exchange. Our results suggest that under anthropogenic forcing the common variability of the fields contributing to the above-mentioned mechanism (AL SLP, Kuroshio Extension net heat flux and SST) will substantially increase. Net heat flux and SST in the Kuroshio Extension are also proxies for the strengthening of North Pacific storms^57^. Anomalous heat flux due to fluctuations in the Kuroshio Extension SST front cause changes in the near surface baroclinicity and the lower levels of the troposphere and may result in further genesis of storms in the region^57,58^.

Furthermore, changes in the atmospheric pressure conditions and the wind patterns over the North Pacific as well as ENSO events have been correlated with the generation of marine heat waves and extreme sea surface temperature anomalies that governed the area in the last decade^59,60^. The intensification of extreme AL may contribute to explaining the prolonged and more frequent presence of marine heat waves in the region over the past years^61^. It may also assist in further understanding and predicting such events, since changes in the sea level pressure have been directly linked to previous marine heatwave events^62^.

An AL extreme deepening has been previously linked to the major marine regime shift in the North Pacific in the late 1970’s^9,10^. Abrupt shifts are predicted to increase in magnitude and consequences under climate change, depending on the severity of the emissions scenario^63,64^. As such, the intensity and frequency of biological shifts in the North Pacific communities could increase in the future, following the late 1970’s example^10^. Such shifts have the potential to significantly affect fishing activities^65,66^ and have profound socio-economic impacts both on regional as well as global scales^67–69^.

Our findings reveal a climate change-induced intensified atmospheric and oceanic variability over the North Pacific, where a strengthening of the AL SLP corresponds to changes in temperature and precipitation patterns and affects the North Pacific oceanic conditions. The potential impacts of such extreme events on the biological and physical conditions of the region stresses the increasing need for continuous monitoring of oceanic conditions, and a rapid advance in our predictive and adaptive capabilities.

## Methods

### CESM1-LENS datasets

The North Pacific region from 20° N to 60° N and 100° E to 90° W is considered here. The CESM1-LENS is specifically designed to provide information on internal climate variability^36^. All the ensemble members use the same model parameters; however, each member represents a distinctive climate trajectory. This is achieved by initializing the simulations with small round-off differences in the air temperature^50^. Here the daily and monthly SLP, surface air temperature, precipitation, sea surface temperature and net heat flux from 36 members of both the historical and RCP8.5 scenario simulations of the CESM1-LENS from 1920 to 2100 are used. Despite the slightly different initial atmospheric conditions, the ensemble members share the same external historical and future RCP8.5 forcing scenarios and use the same model components. Consequently, the resulting uncertainty in the projections is due to internal climate variability alone, giving the advantage of identifying details of processes, such as the PDO^70^. The effects of AL on the long-term North Pacific climate variability^21,34^ highlight the importance of the SLP internal variability, which becomes evident in the multiple simulations of the CESM1-LENS, each of which is forced by an identical scenario of historical and RCP8.5 radiative forcing^42^.

Multiple realizations may also contribute to the understanding of extreme patterns by providing adequate statistical sampling power. The large ensemble size of the CESM1-LENS allows the diagnosis of physical mechanisms for intra-model differences, providing the advantage of accounting for both internal and model variability^36,49^ and assessing the statistics of similar events in different realizations that do not reach full agreement with each other. Strong correlations between modeled and observed patterns in NCAR's Climate Analysis Section diagnostics reveal the realistic representation of the system by CESM1-LENS^71^. Specifically for the North Pacific, large-scale patterns such as the Pacific Decadal Oscillation (PDO) are highly related to the PDO index with an average correlation coefficient of 0.86. Furthermore, the SLP is one of best resolved phenomena with an average pattern correlation coefficient of 0.94 between the ensemble members and the observed time-series^71^. Similarly, the total precipitation and SST are two of the best represented parameters with average correlation coefficients of 0.8 and 0.75, respectively. Detailed information about the CESM1-LENS model can be found in Kay et al.^36^.

### Dynamical indicators

To examine the persistence and predictability of extreme events in both past and future simulations of the CESM1-LENS a dynamical approach is applied to all ensemble members. The idea of the approach is that each state of a system x(t) reaches a point ζ on the attractor and its neighbors are all the other states that have a small Euclidean distance with respect to x(t), defined by a threshold q. Dynamical systems exhibiting chaotic dynamics are characterized by strange attractors, *i.e.* compact geometric objects where the trajectories settle. The existence of attractors ensures the repeatability of a state ζ and the time the dynamics remain in the neighborhood of the state ζ of the system, represented here by the instantaneous dimension $d(\zeta )$ and the inverse persistence θ(ζ) respectively. Specifying these two properties assists in understanding the behavior of the system. These can be estimated by setting a small distance as the threshold, q (2nd and 98th percentile of the time-series) and fitting a Generalized Pareto distribution (GPD) to the tail observations. The approach follows the Peaks Over Threshold method stating that the exceedances above an upper threshold follow a GPD, requiring that the cumulative distribution function of the variable belongs to the max-domain of attraction of the generalized extreme value distribution^72^.

#### Instantaneous dimension

The extreme values laws are used in this approach in order to characterize the point on the attractor: a fixed point ζ on a chaotic attractor presents a probability P that a trajectory $x\left(t\right)$ approaches again the point ζ within a sphere with radius $\varepsilon$ centered on ζ. The Euclidean distance between the state ζ and all other observations of the system is:

1 $$g\left(x\left(t\right)\right)=-{\mathrm{log}}\left(\delta \left(x\left(t\right),\zeta \right)\right)$$

where $\delta \left(x,y\right)$ is the Euclidean distance between two vectors, which tends to zero when x and y are close. The logarithm calculation increases the discrimination of small values of $\delta \left(x,y\right)$, which correspond to large values of $g\left(x\left(t\right)\right)$. The exponential law can describe the probability of logarithmic returns:

2 $$P\left(g\left(x\left(t\right)\right)>q,\zeta \right)\approx exp\left[-\frac{x-\mu \left(\zeta \right)}{\sigma \left(\zeta \right)}\right]$$

where location (µ) and scale (σ) parameters depend on the selected point ζ on the attractor. Specifically, $\sigma \left(\zeta \right)=1/d(\zeta )$, where $d(\zeta )$ is the instantaneous dimension around the point ζ ^73^. Also, q is an upper threshold, and is related to the radius $\varepsilon$ of the trajectory of the system via $q={g}^{-1}\left(\varepsilon \right)$. Requiring that the series of $g\left(x\left(t\right)\right)$ is over the threshold q (percentile selection) is similar to the requirement that the trajectory of the system falls within a sphere around the point ζ. Repeating several iterations for different points ζ makes it possible to obtain the dimension of the attractor:

3 $$D=\overline{ d(\zeta)}$$

where $\overline{d(\zeta )}$ indicates the instantaneous dimensions averaged over all states ζ.

#### Inverse persistence

Estimation of inverse persistence in the phase space assists in testing whether the state ζ is in the neighborhood of a fixed point of the attractor or not. If the system were stuck in the same trajectory ($x\left(t+1\right)=x\left(t\right)$ for all t) for an infinite time, then the previous results for the instantaneous dimensions do not hold. Persistence time can be estimated as an additional parameter in the previous law, the extremal index, θ:

4 $$P\left(g\left(x\left(t\right)\right)\right)>q\approx exp\left[-\theta \frac{x-\mu \left(\zeta \right)}{\sigma \left(\zeta \right)}\right]$$

where θ represents the inverse of the mean residence time within the sphere. Low θ values (close to 0) imply a high persistence of the system, whereas high θ values (close to 1) denote that the trajectory immediately leaves the ζ neighborhood. The value of θ is estimated by using the Süveges maximum likelihood estimator^74^:

5 $$\widehat{\theta }=\frac{\sum_{i=1}^{N-1}\rho {S}_{i}+N-1+{N}_{c}-{\left[{\left(\sum_{i=1}^{N-1}\rho {S}_{i}+N-1+{N}_{c}\right)}^{2}-8{N}_{c}\sum_{i=1}^{N-1}\rho {S}_{i}\right]}^\frac{1}{2}}{2\sum_{i=1}^{N-1}\rho {S}_{i}}$$

where $N$ are the observations exceeding a defined threshold, $\rho$ represents the distribution function for the selected threshold, ${S}_{i}$ is the exceedance distances and ${N}_{c}=\sum_{i=1}^{N-1}I ({S}_{i}\ne 0)$, where $I$ is the indicator function for the selected ${S}_{i}$. For further details on the calculation of the extremal index see ref^74^.

### Cross-wavelet coherence

Multi-scale atmospheric and oceanic variability in the North Pacific may present different spatial and temporal ranges, from local spatial events to multi-decadal temporal patterns, non-stationarity and persistence^1,75,76^. As such, quantifying the relationship between the average monthly Kuroshio Extension net heat flux and AL SLP time-series through classic cross-correlation methods that use a defined time-lag and which assume independence may give spurious results^77^. Here we use cross-wavelet coherence analysis to detect the time–frequency space in which two time-series present high common power^78^. This approach is based on the time-series decomposition via wavelets and presents the association through phase relationships^79^. The Morlet wavelet is used as the ‘mother’ wavelet since it balances in an optimum way the localization both in time and frequency^78^. To estimate the significance level at each frequency, Monte Carlo methods were used, in which the wavelet coherence is calculated for pairs of parameters (i.e. SLP and net heat flux) of a large (order of 1000) surrogate dataset with the same AR(1)^78^. The Morlet wavelet used in the wavelet analysis is defined as

7 $${\psi }_{0}\left(\eta \right)={\pi }^{-{1}/{4}}{ e}^{i{\omega }_{0}\eta } {e}^{-\frac{1}{2}{\eta }^{2}}$$

where ${\psi }_{0}\left(\eta \right)$ is the wavelet function, $i$ is the imaginary unit, ${\omega }_{0}$ is dimensionless frequency and $\eta$ is dimensionless time. The Continuous Wavelet Transform of a time-series ${x}_{n}$ ($n=1,\dots ,N)$ with uniform time steps ${\delta }_{t}$ is the convolution of ${x}_{n}$ with the scaled and normalized wavelet:

8 $${W}_{n}^{X}\left(s\right)=\sqrt{\frac{\delta t}{s}}\sum_{{n^\prime}=1}^{N}{x}_{{n^\prime}}{\psi }_{0}\left[\left({n^\prime}-n\right)\frac{\delta t}{s}\right]$$

where ${\left|{W}_{n}^{X}\left(s\right)\right|}^{2}$ is defined as the wavelet power which can be interpreted as the local phase and represents the variance with respect to the frequencies in the signal, ${\psi }_{0}$ is the normalized wavelet, $s$ is the wavelet scale, $n$ is the localized time index, and ${n^\prime}$ the translated time index of the time ordinate $x$. ﻿The global power refers to the time integration of all the local wavelet spectra, if we considered a vertical slice through the wavelet plot as a local measure of the spectrum^80^. The global wavelet power is defined as:

9 $${\overline{W}}^{2}\left(s\right)=\frac{1}{N} \sum_{n=0}^{N-1}{\left|{W}_{n}\left(s\right)\right|}^{2}$$

Cross wavelet coherence is analogous to the correlation coefficient in a specified spatial and temporal frequency space

10 $${R}_{n}^{2}\left(s\right)=\frac{{\left|S \left({s}^{-1}{W}_{n}^{XY}\left(s\right)\right)\right|}^{2}}{S\left({s}^{-1}{\left|{W}_{n}^{X}\left(s\right)\right|}^{2}\right)\cdot S\left({s}^{-1}{\left|{W}_{n}^{Y}\left(s\right)\right|}^{2}\right)}$$

where S is a smoothing operator in the scale axis and time domain, and it is defined as

11 $$S\left(W\right)={S}_{scale}\left({S}_{time}\left({W}_{n}\left(s\right)\right)\right)$$

where ${S}_{scale}$ and ${S}_{time}$ describe smoothing along the wavelet scale and time axes respectively, which should have a similar form to the mother wavelet, the Morlet wavelet here^81^. The statistical significance of the wavelet coherence is tested with Monte Carlo methods^78^.

## Supplementary Information

Supplementary Information.
