# Supplementary material for: Future intensification of extreme Aleutian low events and their climate impacts
Source: Sci Rep. 2021 Sep 15;11:18395. doi: 10.1038/s41598-021-97615-7 (PMC8443677; doi:10.1038/s41598-021-97615-7)
Supplement: Supplementary file 1 — Supplementary Information. [file 41598_2021_97615_MOESM1_ESM.docx]

# Supplementary Information

## Intensification of future Aleutian Low extremes

To capture the evolution of the AL SLP in the future we compared the spatial average of monthly AL SLP of the historical simulations to the future RCP8.5 runs. Figure S1 presents a comparison between the spatially averaged time-series of the AL SLP (Figure S1a) and its extreme low conditions (Figure S1b) in the past and future ensemble members. The extreme low AL SLPs were defined as the 2% percentiles of both ensemble members, which are significantly different (Rank-sum test, p-value < 0.05; Figure S1). This result suggests a generalized deepening of the low SLP in the future compared to the past simulations, *i.e.* a strengthening of the AL under anthropogenic warming. Gan et al. ^34^ calculated the future North Pacific Index using a multi-model average in the 21^st^ century, showing an intensification of the AL in the future. Various modeling studies proposed the intensification and increasing frequency of ENSO under the RCP8.5 scenario ^30,31,32,33,35^, and therefore an increase of AL extreme events is anticipated.


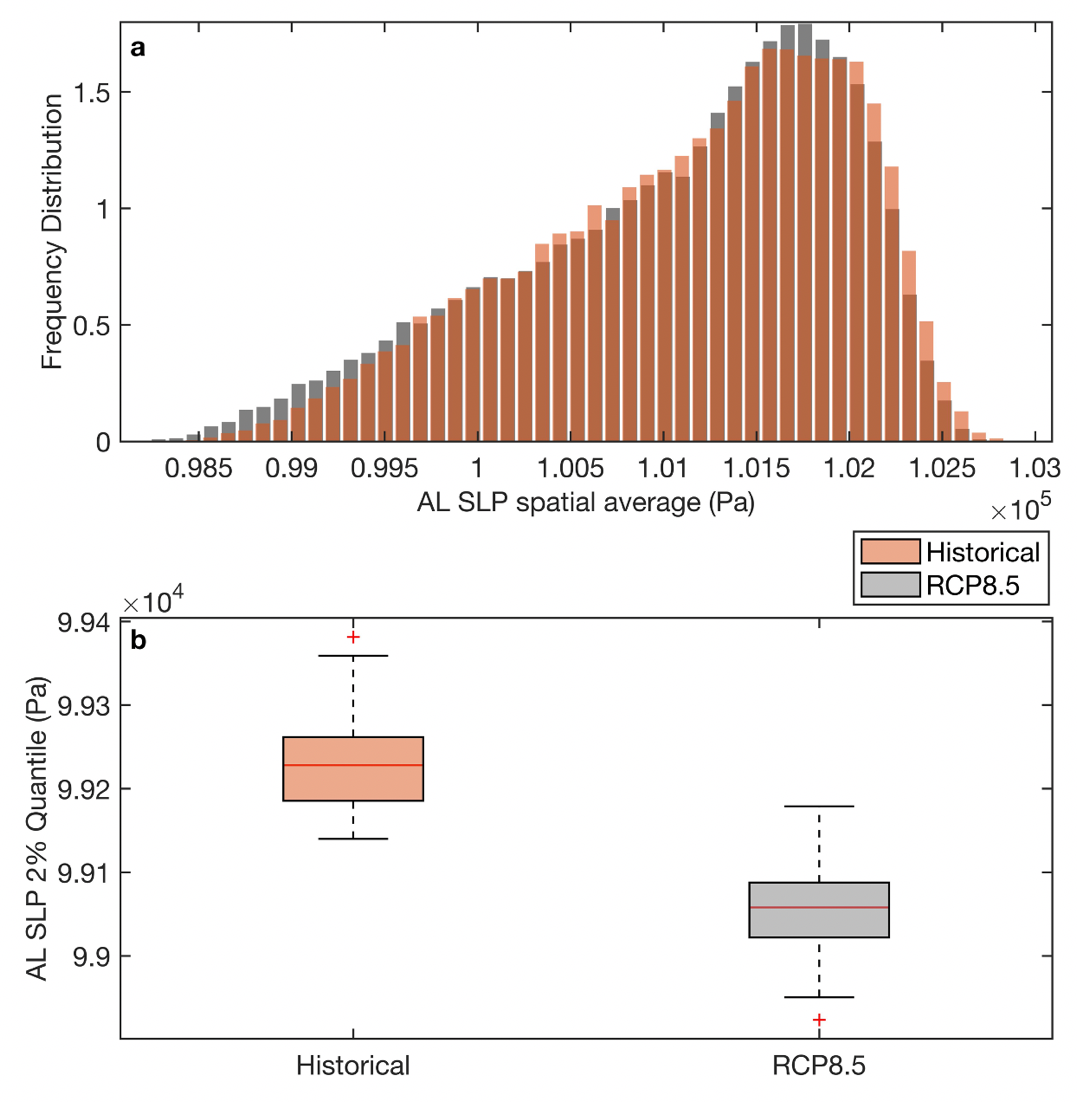


Figure S1: (a) Frequency distribution of the spatially averaged SLP in the AL area covering the box of 45° -60° N latitude and 150° -210° longitude for the total of the historical (grey) and RCP8.5 future (orange) simulations. (b) Boxplots of the lower 2% quantile threshold of the spatially averaged AL SLP time-series of each ensemble member (n=30). The red lines indicate the medians for each ensemble scenario.

**Correlations between the AL SLP and weather parameters**

Spearman correlation coefficients between the AL SLP and point-wise SAT/precipitation over North America were calculated separately for the historical and the RCP8.5 simulations in order to reveal the spatial relationship of the AL and weather parameters. Figures S2 and S3 present the ensemble average correlation coefficients. The patterns and the intensification of negative correlations between the examined parameters (AL SLP and SAT/precipitation) that are revealed here, allowed us to proceed to the application of wavelet coherence in order to further examine these relationships in different frequencies.


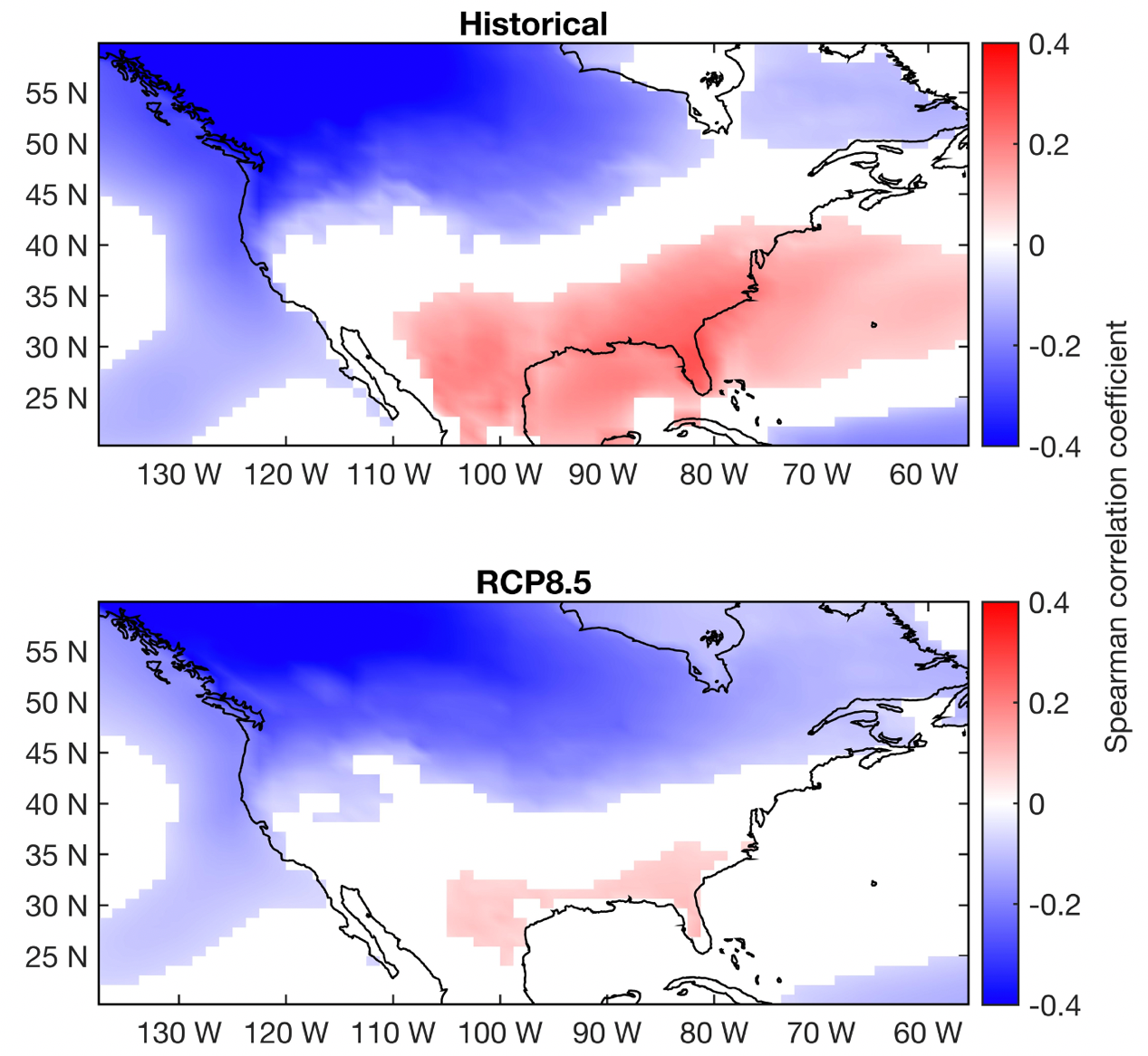


Figure S2: Point-wise correlation coefficient between the spatially averaged AL SLP and SAT in north America (a) for historical and (b) RCP8.5 simulations.


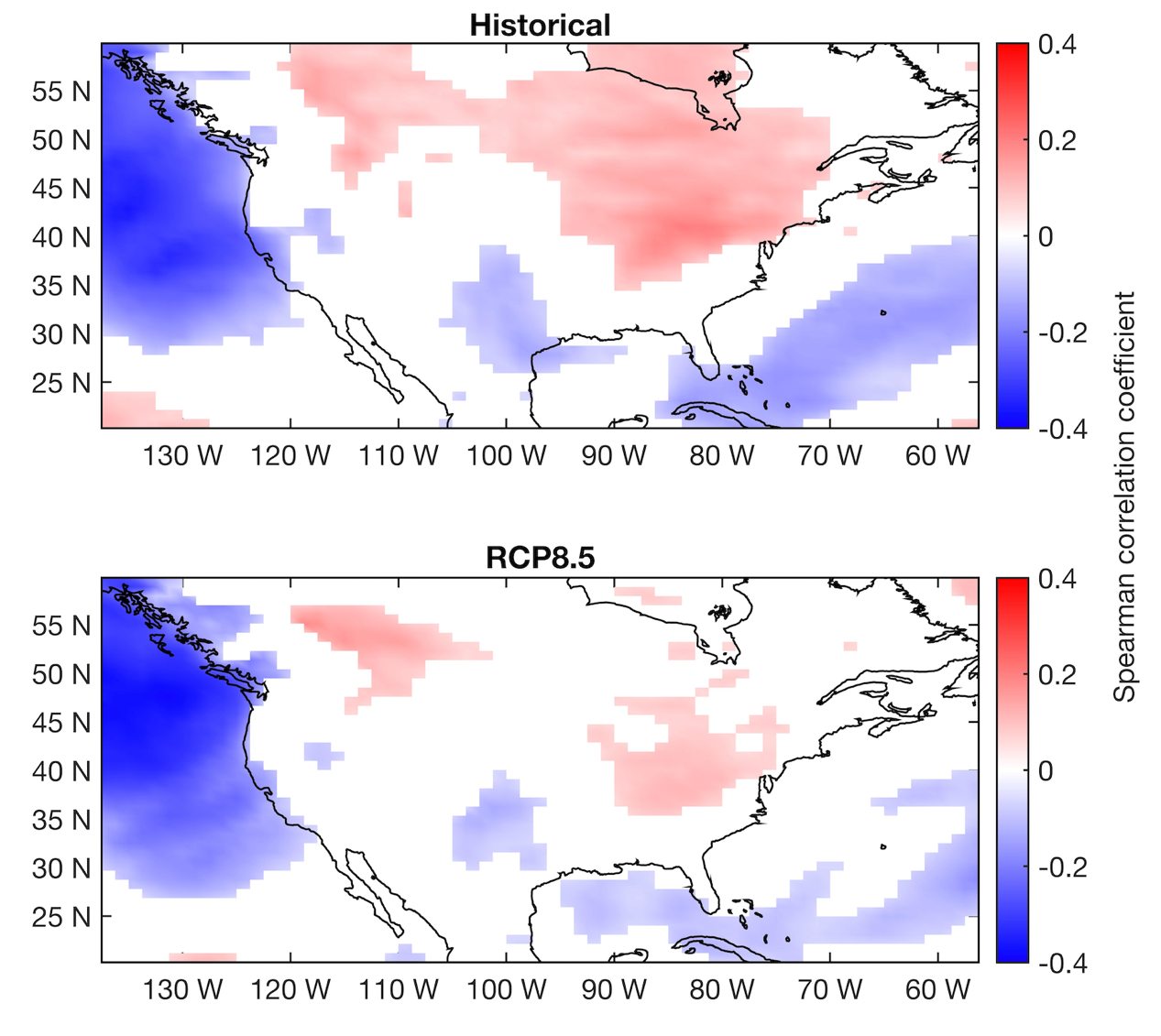


Figure S3: Point-wise correlation coefficient between the spatially averaged AL SLP and precipitation over north America (a) for historical and (b) RCP8.5 simulations.

**Empirical Cumulative Distribution Functions**

The Empirical Cumulative Distribution Functions (ECDFs; Figure S4) were calculated here at the 99% significance level, in order to support the two-sided Kolmogorov-Smirnov test on whether the data come from different distributions.


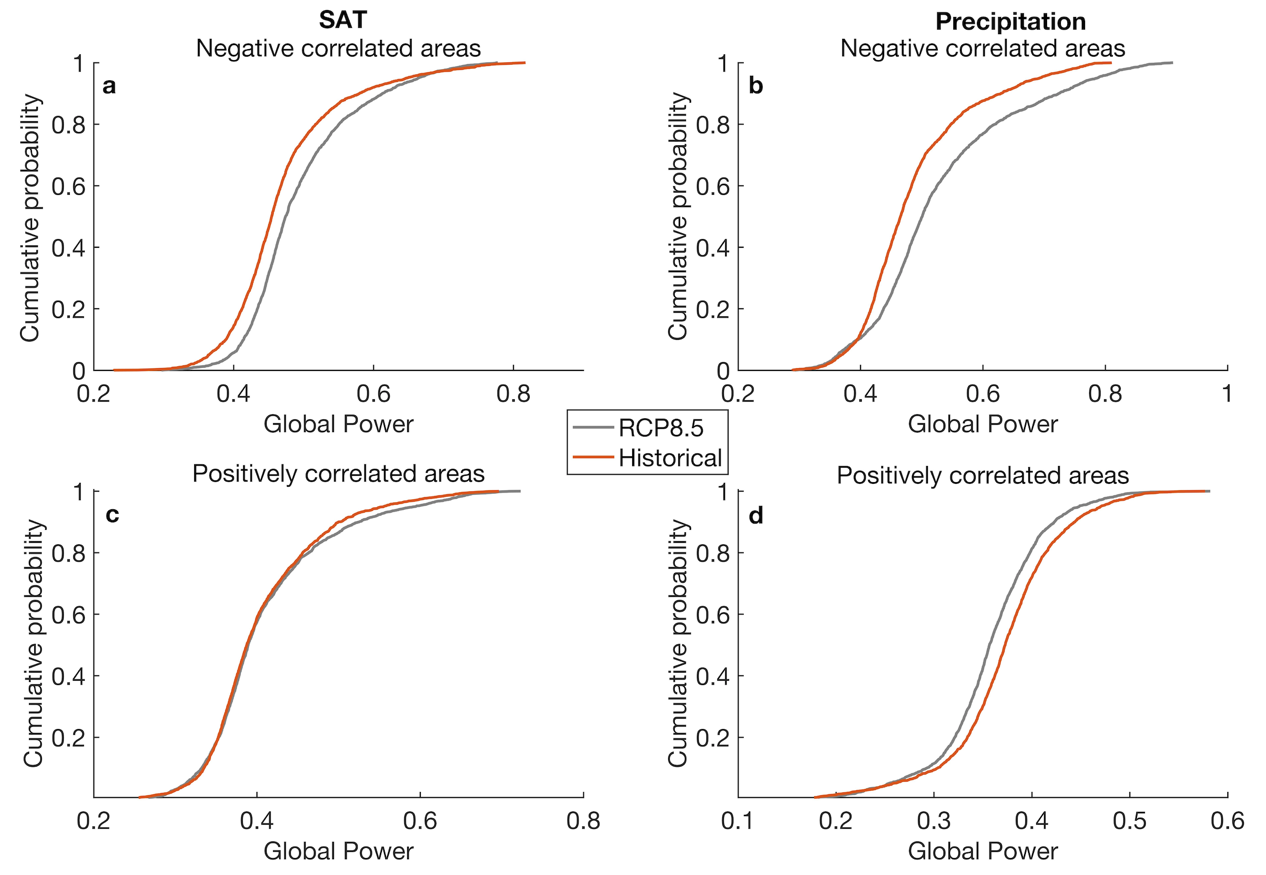


Figure S4: Empirical Cumulative Distribution Functions for the global power of the wavelet coherence calculated for the historical and future CESM-LENS simulations between the AL SLP and the SAT on (a) the negatively and (c) the positively correlated areas and the AL SLP and the precipitation on (b) the negatively and (d) the positively correlated areas.

## Relationship between Aleutian Low and climate parameters on different timescales

In addition to the comparison between historical and full RCP8.5 time-series, alternative timescales have also been tested in order to identify whether the SLP pattern over the Aleutians exhibit different characteristics in the near- and far- future periods. The persistent El Nino like SST may be more prominent in the latter half of the century, therefore the examined RCP8.5 time series have been separated into two periods of 2005-2050 and 2051-2100. Indeed, the global power of the cross-wavelet coherence calculated for the far future (2051-2100) simulations and the different climate parameters (net heat flux and SST in the Kuroshio extension, precipitation and air temperature over North America) in most cases is presented to be increased compared to the near future (2005-2050) power. An exception is presented in the global power of the positive correlated areas of precipitation oven North America and the AL time series (Figure S6b), in which the far future simulations have lower power compared to the historical and near future runs.


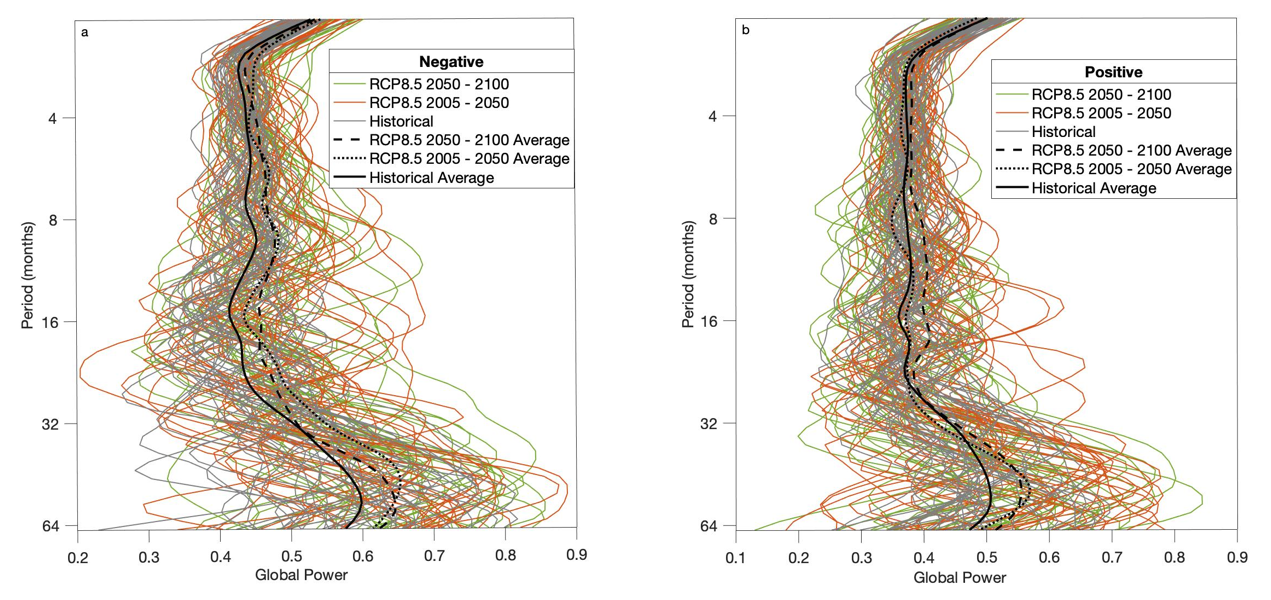


Figure S5: Cross-wavelet coherence of the negative (a) and positive (b) correlated areas between the AL and air temperature over North America time series of the historical, RCP8.5 over the period 2005-2050 and RCP8.5 from 2051 to 2100.


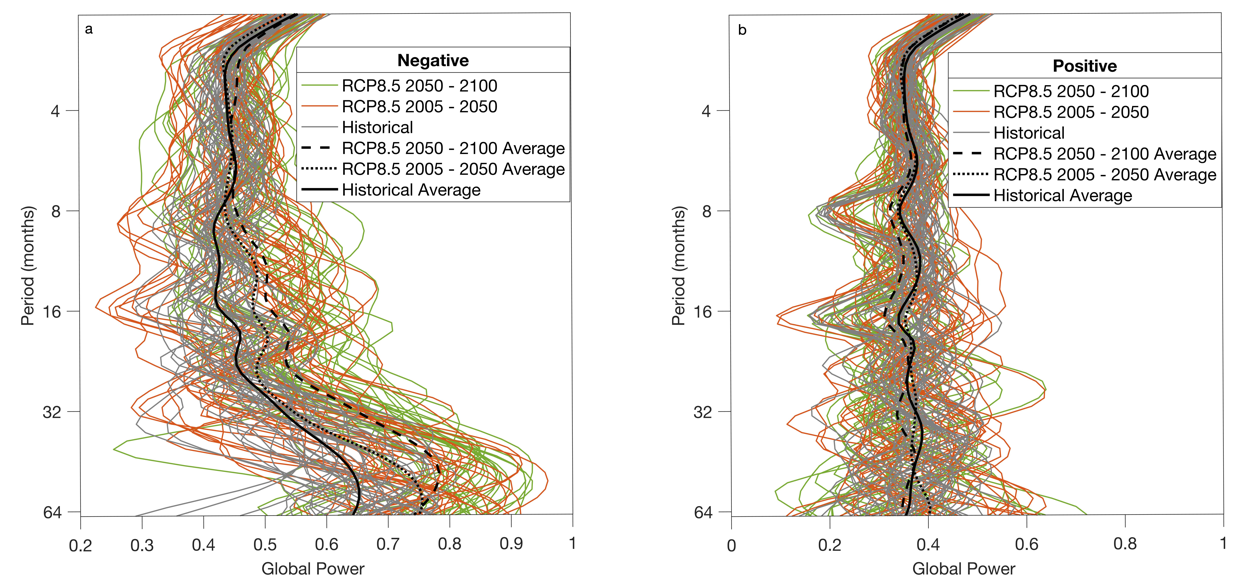


Figure S6: Cross-wavelet coherence of the negative (a) and positive (b) correlated areas between the AL and precipitation over North America time series of the historical, RCP8.5 over the period 2005-2050 and RCP8.5 from 2051 to 2100.


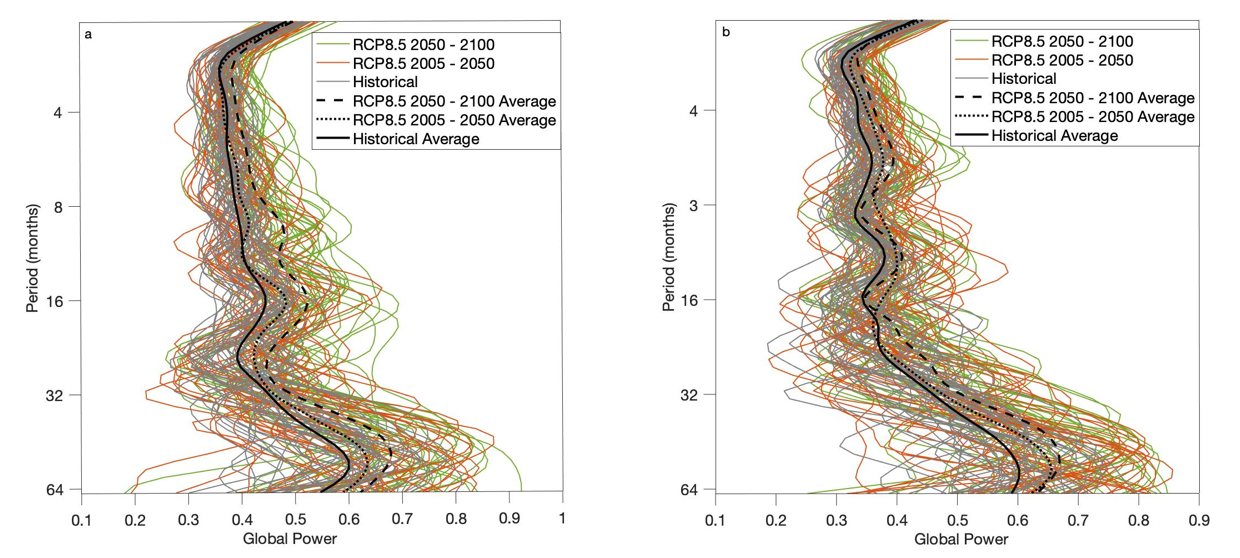


Figure S7: Cross-wavelet coherence between the AL and (a) SST and (b) net heat flux time series in the Kuroshio extension area for the historical, RCP8.5 over the period 2005-2050 and RCP8.5 from 2051 to 2100.

## CESM-LENS validation against reanalysis time-series

In order to support the validity of our analysis, a comparison of CESM-LENS model output against the reanalysis timer-series is presented. Daily and monthly SLP, SAT, total precipitation, SST and net heat flux reanalysis data were obtained from the National Center for Environmental Prediction/National Center for Atmospheric Research (NCEP/NCAR) reanalysis project ^79^ with spatial resolution of 2.5° x 2.5° from 1949 to 2018. Figure S8 presents the comparison between the North Pacific monthly anomalies time-series of both the reanalysis data and all the 36 ensemble members of the historical simulations of CESM1-LENS for the period 1949-2005. The model is shown to closely follow the SLP observational data. However, it overestimates or underestimates the rest of the variables at different times throughout the time-series.


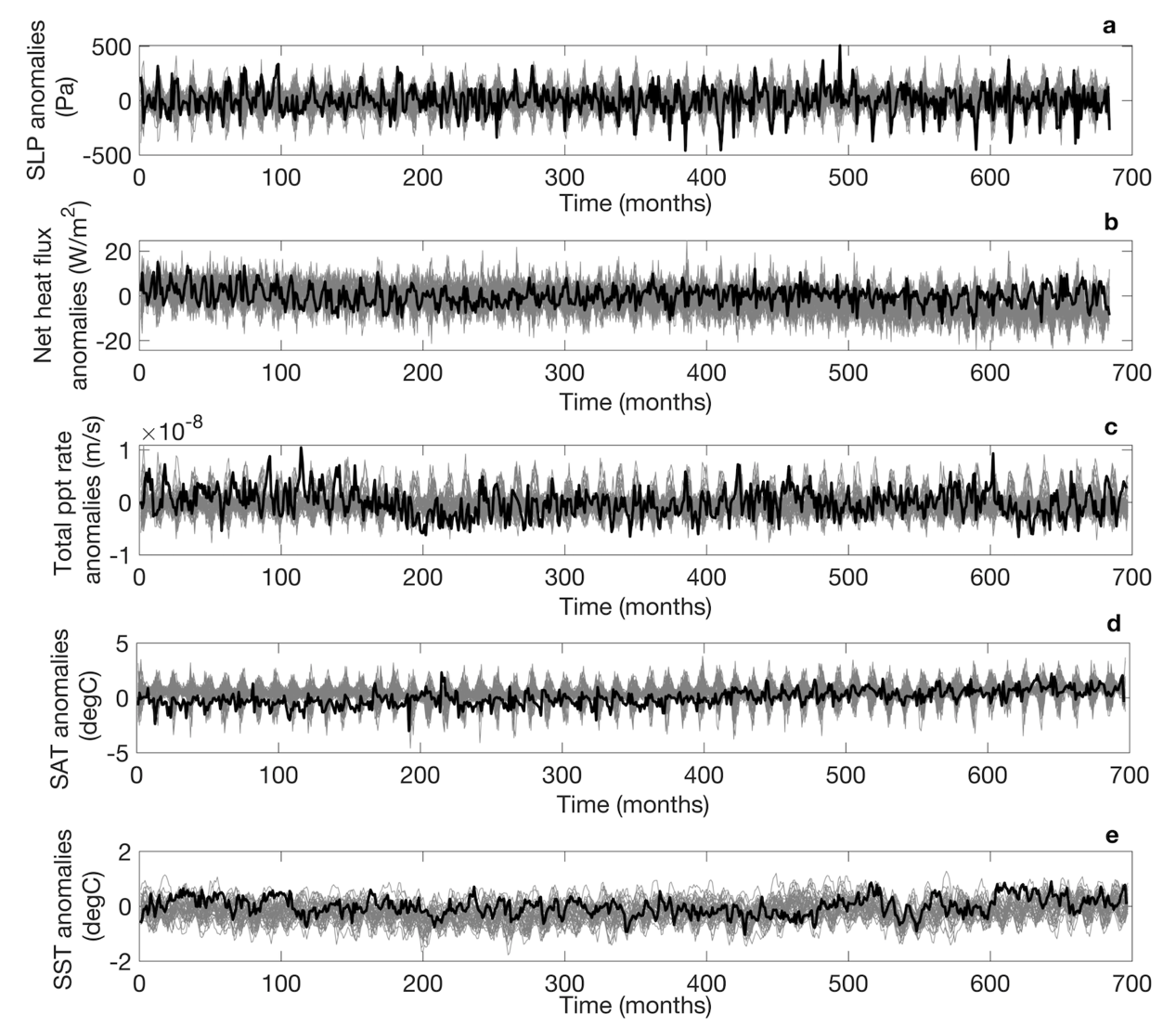


Figure S8: Comparison between monthly reanalysis time-series and historical simulations CESM-LENS output for (a) SLP, (b) net heat flux, (c) total precipitation rate, (d) SAT and (e) SST.
